# Dining in danger: Resolving adaptive fish behavior increases realism of modeled ecosystem dynamics

**DOI:** 10.1002/ece3.70020

**Published:** 2024-08-07

**Authors:** Nicolas A. Schnedler‐Meyer, Tobias K. Andersen

**Affiliations:** ^1^ National Institute for Aquatic Resources Technical University of Denmark Lyngby Denmark; ^2^ Institute for Ecoscience Aarhus University Aarhus Denmark

**Keywords:** adaptive behavior, behavioral cascade, ecosystem modeling, food web, lake ecology, nonconsumptive effects, predation, trophic cascade

## Abstract

Animals occupying higher trophic levels can have disproportionately large influence on ecosystem structure and functioning, owning to intricate behavioral responses to their environment, but the effects of behavioral adaptations on aquatic ecosystem dynamics are underrepresented, especially in model studies. Here, we explore how adaptive behavior of fish can affect the dynamics of aquatics ecosystems. We frame fish behavior in the context of the central trade‐off between feeding and predation, calculating the optimal level of feeding determined by ambient food availability and predation risk. To explore whole‐ecosystem consequences of fish behavior, we embed our behavioral model within the Water Ecosystems Tool (WET), a contemporary end‐to‐end aquatic ecosystem model. The principle of optimality provides a robust and mechanistic framework for representing animal behavior that is relevant for complex models, and can provide a stabilizing effect on model dynamics. The model predicts an emergent functional response similar to Holling type III, but with richer dynamics and a more rigorous theoretical foundation. We show how adaptive fish behavior works to stabilize food web dynamics compared to a control model with no optimal behavior, and how changing the strength of the underlying trade‐off has profound effects on trophic control and food web structure. Furthermore, we demonstrate how including fish behavior allows for an overall more realistic response of the model system to environmental perturbation in the form of nutrient enhancement. We discuss the structuring effects of behavioral adaptations in real ecosystems, and how approaches like this one may benefit aquatic ecological modeling. Our study further highlights how a mechanistic approach based on concepts from theoretical ecology can be successfully implemented in complex operational models resulting in improved dynamics and descriptive power.

## Graphical abstract

We investigated how the trade‐off between predator avoidance and feeding in fish can influence the function and structure of a modeled lake ecosystem. The results showcase how more realistic behavior in ecosystem models can have many benefits, including more realistic model responses to perturbation and increased model stability.

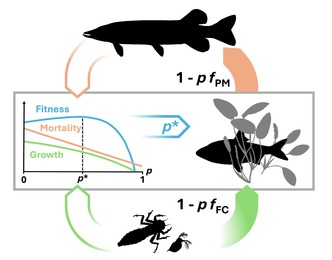

## 1 INTRODUCTION

Animals constantly adapt their behavior in response to a changing environment. In particular, most animals must constantly face decisions on how to forage, how much to forage, and on what, based on information from environmental variables such as food availability and predation pressure, and these decisions can have far‐reaching consequences for ecosystems and their dynamics (e.g., Beckerman et al., 2019; Brown et al., 1999; Ripple & Beschta, 2012; Schmitz, 2008). In this way, the impact of an animal population on the surrounding ecosystem can vary both qualitatively and quantitatively, even with no change in population size. Indeed, for larger animals, behavioral adaptations and their associated consequences for the surrounding food web can change at much shorter time scales than populations and biomasses (Brown et al., 1999). Accordingly, larger animals with complex behaviors can play an important structuring role in food webs, engaging in complex feedback loops that can both promote and impair the successful management of ecosystems (e.g., Brown et al., 1999; Carpenter et al., 1985; Folke et al., 2004; McArthur et al., 2014; Ripple & Beschta, 2012; Schmitz, 1998; Schmitz et al., 2000). A well‐known example of this occurs in lake ecosystems, where fish community structure plays a large role in determining structure and composition of the ecosystem as a whole, despite their relatively low contribution to total lake biomass (Jeppesen et al., 1997; Scheffer & Jeppesen, 2007).

However, studies of aquatic ecosystem have traditionally tended to focus on small or microscopic planktonic organisms, which is reflected in the models simulating aquatic ecosystem dynamics (Menshutkin et al., 2014; Mooij et al., 2010; Shin et al., 2010). Such organisms do often dominate the total productivity of aquatic ecosystems and respond much more directly to environmental changes. Perhaps for these reasons, behavioral responses have not been common in complex ecosystem models, not even in models that include organisms at higher trophic levels. Additionally, ecological effects of behavior of especially larger animals are difficult to study, both in situ and in the laboratory. Therefore, models remain our best tool for exploring the potential consequences of animal behavior for ecosystem dynamics.

Most traditional process‐based ecosystem models feature static descriptions of population dynamics and trophic interactions—the assumption that animals are always feeding at the maximum capacity dictated by the environment, and that trophic interactions are therefore also always at maximum strength (see, e.g., Ahrens et al., 2012; Gentleman et al., 2003; Power, 1992; Visser & Fiksen, 2013 for discussions of these common assumptions). This means that populations can only respond numerically to environmental fluctuations, which induces instability in the population dynamics, especially for organisms with long generation times and relatively low vital rates. Furthermore, when trophic coupling is assumed tight and inflexible, trophic control is predicted to be strong, resulting in strong top‐down trophic cascade patterns in ecosystem structure with increasing system productivity, at least in systems with no additional density‐dependence (Heath et al., 2014; Oksanen et al., 1981). However, most empirical patterns of ecosystem structure across productivity gradients do not follow this pattern exactly, instead showing varying and dynamic trophic interaction strength, which is reflected in various combinations of so‐called bottom‐up and top‐down responses (Heath et al., 2014; Jeppesen et al., 2000; Power, 1992). Rigid, too‐strong trophic control in models are often alleviated by the inclusion of density‐dependence to the equations governing the population dynamics, in the form of carrying capacities or quadratic mortality terms (Geritz & Kisdi, 2012; Heath et al., 2014). However, such measures are not ecologically well defined, and consequently hard to measure, quantify and interpret, and it has been argued that it is favorable to avoid such abstractions if they can be replaced at a lower, more mechanistic level (Geritz & Kisdi, 2012; Pinti et al., 2021).

Numerous lines of research support the notion that fish (and indeed most other animals) do not maximize feeding at all times, but continuously trade‐off feeding against other determinants of fitness, such as mortality, metabolic costs, and reproduction (Biro et al., 2003, 2006; Fraser & Gilliam, 1987; He & Kitchell, 1990; Pettersson & Brönmark, 1993; Romare & Hansson, 2003; Schmitz et al., 2008). Animals continuously balance trade‐offs related to energy costs, feeding, and mortality by rapidly changing behavior, for example, by responding to increases in food availability or predation pressure with diet switches or behaviors that lower predation risk (Lima & Bednekoff, 1999). Such risk‐reducing behaviors include use of habitat restrictions or refuge habitat, diel or general activity decreases, and flocking or schooling behavior, and may in turn promote behavioral shifts further down the food chain (a so‐called behavioral cascade, e.g., Romare & Hansson, 2003). These behavioral responses can have stabilizing effects on population and ecosystem dynamics, through the induction of ratio‐dependent predation rates (Power, 1992), compared to predation that is only dependent on prey availability, as is most often assumed in models.

Better representation of animal behaviors in generalized models could therefore help to stabilize model behavior, while also describing trophic interactions in a more dynamic and realistic way, but such representation is not trivial to implement, given the vast complexity and diversity of observed behavior. However, if seen in an evolutionary frame, all successful behaviors must obey the same evolutionary principles, that is, to find the optimal balance between short‐term growth and reproduction on one side, while minimizing costs and risks on the other, in order to maximize lifetime fitness (Brown et al., 1999; Fraser & Gilliam, 1987; Kiørboe, 2011; Kiørboe et al., 2017; Sainmont et al., 2013). Given strong selective pressure and a relatively stable or predictable environment, it may be argued that animals can be expected to behave optimally with respect to this trade‐off (Perry & Pianka, 1997). This assumption of optimality in behavior has, though not universally accepted, been shown to be a useful framework within which to describe, explain, and predict animal behavior (Biro et al., 2006; Brönmark et al., 2008; Brown et al., 1999; Pettersson & Brönmark, 1993; Ripple & Beschta, 2012; Visser & Fiksen, 2013).

Here, we aim to explore how adaptive fish behavior affects the dynamics of the entire ecosystem, in which they are embedded. To this end, we develop and characterize a deliberately simple model of fish behavior, embedded in the end‐to‐end model WET (Water Ecosystems Tool, Schnedler‐Meyer et al., 2022). Our model formulation is based on the fundamental assumption that fish can lower their predation risk by a range of behaviors, at the cost of decreased feeding efficiency. We intentionally design the model to be agnostic to the specifics of these behaviors but incorporate parameters that can be used to tune the strength of the trade‐off between feeding and predation. We show that by letting fish optimize their behavioral responses to environmental fluctuations, general model behavior may improve, including more stable and realistic population dynamics, and more realistic regulation of the degree of trophic control exerted by fish on lower trophic levels.

## 2 METHODS

### 2.1 Optimal behavior model

We assume fish to be well informed about food availability and predation risk, and to be able to adapt their behavior to maximize their short‐ and long‐term fitness. Our formulation is based on the approach of Kiørboe et al. (2017), with some modifications allowing for the regulation of the strength of the underlying trade‐off. Most implementations of optimal foraging assume two discrete foraging states, separated in space (a foraging arena and a refuge) and/or time (actively feeding/not feeding): either animals are feeding maximally, at the cost of exposing themselves to predators, or they are not feeding at all, in which case predation is also zero. Here, we aim to soften the assumption of a purely discrete choice between feeding and predation, to explore the impact of a more continuous trade‐off, where both the cost (in terms of feeding), as well as the benefit (lowered predation risk) of predation mitigation behaviors can be varied. As a null model, and for the sake of model simplicity, we will assume linear relationships between predation mitigation, and feeding efficiency and predation risk.

Let *p* be a number between zero and one, representing a point on a gradient in average fish behavior along a linear trade‐off of behaviors that goes from maximizing gains at the cost of increasing energy expenditure and predation risk (*p* equals zero), to minimizing costs at the general expense of feeding efficiency (*p* equals one). Now let the strength of the trade‐off along this behavioral gradient be determined by three parameters, the predation mitigation efficiency (*f*_PM_), the feeding cost of predation mitigation (*f*_FC_), and extra metabolic cost associated with feeding (*f*_RF_), along with ambient conditions (food availability and predation risk). At *p* = 0, fish consumption is the maximum consumption dictated by food availability and feeding parameters (no time spent on predation mitigation), while at *p* = 1, this consumption is reduced according to *f*_FC_ (between zero and one). Under normal conditions (i.e., when net energy gain from feeding is positive), the current feeding activity of the fish population is thus given by:

(1) $$f_{\text{Feed}}=1-pf_{\mathrm{FC}}$$

Following Kiørboe et al. (2017), we assume foraging to be mainly a gut‐limited process (Armstrong & Schindler, 2011; Fall & Fiksen, 2020; Jeschke et al., 2002), in which case $f_{\text{feed}}$ modulates the effective clearance rate with prey, and therefore also satiation of the predator. The WET fish module supports a range of fish feeding modes and diet compositions. See the next section for an overview of how these are implemented, as well as Appendix S1 for a detailed formulation of the entire feeding algorithm. For now, assuming a simple case with only a single prey type, the feeding level of the fish at a given value of *p*, can be calculated as:

(2) $$F\left(p\right)=\frac{f_{\text{Feed}}f_{\mathrm{veg}}\;cf_{\mathrm{gut}}R\;}{I_{\max}+f_{\text{Feed}}f_{\mathrm{veg}}cf_{\mathrm{gut}}R}$$

where c is the maximum clearance rate, $I_{\max}$ is (temperature‐dependent) maximum (gut‐limited) consumption, *f*_gut_ is the gut occupation factor (higher for prey of low digestibility or energy density), and $f_{\mathrm{veg}}$ is the (optional) impact of vegetation on catchability, calculated dynamically in each time step (see Appendix S1). Ingestion *g* is then calculated as total encountered and caught prey, times the “hunger level” (one minus the feeding level), times the assimilation efficiency a:

(3) $$g\left(p\right)={\mathrm{af}}_{\text{feed}}f_{\mathrm{veg}}\mathrm{cR}\left(1-F\right)$$

This formulation of the functional response is identical to a Holling type II when $f_{\text{Feed}}$ = 1, with $f_{\mathrm{gut}}/I_{\max}$ and $f_{\mathrm{veg}}c$ corresponding to the handling time and attack rate of a classic Holling type II formulation, respectively. Our formulation is preferable in case of multiple prey, as it allows for distinction in quality and clearance rates among prey.

The vulnerability to predation when predation mitigation is maximized by the fish (*p* = 1) is given by the predation mitigation efficiency *f*_PM_ (between zero and one), such that total mortality of the fish at any given value of *p* is:

(4) $$m_{\mathrm{tot}}\left(p\right)=m_{\mathrm{nat}}+\left(1-pf_{\mathrm{PM}}\right)m_{\text{pred}.s}$$

where *m*_nat_ is the background natural mortality, and *m*_pred.s_ is the smoothed out maximum predation mortality (at *p* = 0), obtained by summing up the current population‐level clearance rates of all predators. In order to prevent erratic switching back and forth of prey and predator feeding efforts, and to compensate for unrealistically short reaction times for small time steps, predation mortality fluctuations are smoothed out by the following function:

$$m_{\text{pred}.s}=\frac{m_{\text{pred}.s.\text{prev}}+0.25m_{\text{pred}}}{1.25}$$

where *m*_pred.s_ is the smoothed out predation mortality in the current time step, *m*_pred.s.prev_ is the smoothed out predation mortality of the previous time step, and *m*_pred_ is the current instantaneous predation rate. Finally, we assume that foraging is associated with metabolic costs related to the feeding process, such that total energy expenditure by fish is:

(5) $$u_{\mathrm{tot}}\left(p\right)=\left(1+f_{\mathrm{RF}}f_{\text{feed}}\right)u$$

where *f*_RF_ is the extra metabolic cost associated with feeding, relative to the basal (temperature‐dependent) metabolic rate u.

**FIGURE 1:**
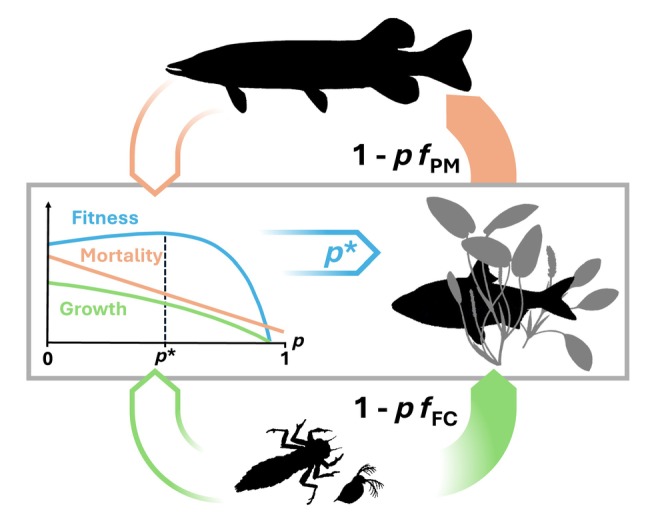
Graphical representation of the optimal behavior model. Biomass transfer between trophic levels depends on intermediate predator behavior (center right) along a trade‐off between feeding (in green) and predation (in orange). Both predation risk and feeding rate (filled arrows) decrease with the level of risk mitigation employed (*p*), in proportion to trade‐off strength parameters *f*_PM_ and *f*_FC_. Based on current predation risk and prey availability (hollow arrows), fitness of different levels of p can be estimated (blue curve) as growth divided by mortality. The optimal level of risk mitigation (p*) is the one that maximizes fitness.

For any given combination of ambient food availability and predation risk, there will be an optimal value of *p* on the behavioral gradient (Figure 1). As a criterion for evaluating how a given level of *p* influences general fish fitness, we will follow the methodology of Kiørboe et al. (2017) and use Gilliam's rule (Fraser & Gilliam, 1987), which uses net energy gain over mortality as a fitness proxy. This fitness proxy is equal to the lifetime reproductive output in a stale environment, and a good estimate in varying environments (Sainmont et al., 2015). In the special case where net energy gain is negative, we can disregard predation because the immediate concern is minimizing losses due to starvation, and in this case, prolonging the time to starvation death by maximizing net energy gain is assumed optimal (Kiørboe et al., 2017).

The optimal value of *p* that maximizes our fitness criterion *W* (hereafter denoted as p*) can therefore be found by maximizing the following fitness function:

(6) $$W\left(p\right)=\left\{\begin{array}{c} \frac{g\left(p\right)-u_{\mathrm{tot}}\left(p\right)}{m_{\mathrm{tot}}\left(p\right)},\;\text{for}\;g_{\mathrm{pot}}-u_{\mathrm{pot}}\geq 0\\ g\left(p\right)-u_{\mathrm{tot}}\left(p\right),\;\text{for}\;g_{\mathrm{pot}}-u_{\mathrm{pot}}<0 \end{array}\right.,$$

where $u_{\mathrm{pot}}$ and $g_{\mathrm{pot}}$ are the total potential metabolic cost and the potential assimilation rate, respectively, when assuming maximized feeding (i.e., *p* = 0). We will deal with the special case where net energy gain is negative first, as in this case finding p* is trivial; if the net energy gain of constant feeding is negative, but higher than the basic metabolic costs, then fish will maximize time to starvation by maximizing feeding, that is:

(7) $$p*=0,\text{for}\;0>g_{\mathrm{pot}}-u_{\mathrm{pot}}>u$$

However, when food becomes so scarce that the cost of feeding becomes higher than the gains, fish should stop feeding, and are consequently free to maximize their predator avoidance, that is,

(8) $$f_{\text{feed}}=0,p*=1,\text{for}\;g_{\mathrm{pot}}-u_{\mathrm{pot}}\leq u$$

Finally, in the most common case when net energy gain is equal or higher than zero, p* can be found by setting the fitness function equal to zero, and solving for *p*, with the constraint that:

(9) 0≤p*≤1

Using Mathematica (Wolfram Research, Inc; version 13) software to solve Equations (1), (2), (3), (4), (6) analytically, we arrive at the following expression for p*:

$$p^{*}=$$

(10) $$\frac{\begin{array}{c} f_{\mathrm{FC}}f_{\mathrm{gut}}I_{\max}f_{\mathrm{PM}}{R_{e}}^{2}m_{\text{pred}}+uf_{\mathrm{FC}}f_{\mathrm{gut}}R_{e}\left(I_{\max}+f_{\mathrm{gut}}R_{e}\right)\left(f_{\mathrm{FC}}\left(m_{\mathrm{nat}}+m_{\text{pred}}\right)f_{\mathrm{RF}}-f_{\mathrm{PM}}m_{\text{pred}}\left(1+f_{\mathrm{RF}}\right)\right)\\ -\sqrt{{f_{\mathrm{FC}}}^{2}f_{\mathrm{gut}}{I_{\max}}^{2}f_{\mathrm{PM}}{R_{e}}^{2}\left(\begin{array}{c} I_{\max}f_{\mathrm{PM}}R_{e}m_{\text{pred}}\left(f_{\mathrm{FC}}\left(m_{\mathrm{nat}}+m_{\text{pred}}\right)-f_{\mathrm{PM}}m_{\text{pred}}\right)+\\ u\left(f_{\mathrm{FC}}f_{\mathrm{gut}}R_{e}\left(m_{\mathrm{nat}}+m_{\text{pred}}\right)-f_{\mathrm{PM}}m_{\text{pred}}\left(I_{\max}+f_{\mathrm{gut}}R_{e}\right)\right)\\ \left(f_{\mathrm{PM}}f_{\mathrm{RF}}\left(m_{\mathrm{nat}}+m_{\text{pred}}\right)-f_{\mathrm{PM}}m_{\text{pred}}\left(1+f_{\mathrm{RF}}\right)\right) \end{array}\right)} \end{array}}{{f_{\mathrm{FC}}}^{2}f_{\mathrm{gut}}{R_{e}}^{2}\left(I_{\max}f_{\mathrm{PM}}m_{\text{pred}}+uf_{\mathrm{gut}}\left(f_{\mathrm{FC}}f_{\mathrm{RF}}\left(m_{\mathrm{nat}}+m_{\text{pred}}\right)-f_{\mathrm{PM}}m_{\text{pred}}\left(1+f_{\mathrm{RF}}\right)\right)\right)}$$

where $R_{e}$ is encountered prey, that is, the product of R, c, and $f_{\mathrm{veg}}$.

### 2.2 Beyond a single prey type

In the simple case of a single prey type, fish feeding is calculated as in the previous section. However, the WET fish module supports a range of fish feeding modes and diet compositions, the inclusion of which imposes additional steps on the estimation of p*, as well as on the calculation of the overall feeding rate.

This manuscript is primarily concerned with the choice of overall feeding effort. However, in the following, we will give a brief overview of the methodology of determining diet composition and feeding mode selection, and a detailed formulation of the entire feeding algorithm is included in Appendix S1.

Fish feeding decisions in WET are separated into three distinct choices; the choice between feeding modes (pelagic, benthic, and piscivorous), which are assumed to be discrete events in space and time, and thus mutually exclusive; the choice of which available prey types within each feeding mode to include in the diet at any given moment; and the choice of overall feeding effort (or predation mitigation, see previous section). Thus, we assume that the decisions of foraging effort, feeding mode, and diet composition are made independently. The reasoning behind this is mainly that weighing different feeding modes against each other in the context of not only relative feeding rates, but also relative exposure to predation would require precise and detailed knowledge of the associated costs of different feeding modes. Having such detailed knowledge of the study system is not realistic in most applications. Furthermore, including dynamic and changing diets directly into the analytical solution for p* is not possible, due to the iterative nature of determining optimal diet breadth. In each time step, the decisions about predation mitigation (*p*) level, optimal diet composition, and allocation of foraging effort between feeding modes are therefore separated into two steps. First, the diet composition and feeding mode allocation is calculated for all feeding modes, assuming maximized feeding (no predation mitigation), and then these are used as input for the optimal behavior model (OBM, previous section).

The allocation of feeding time between the feeding modes that are available to a fish species in WET follows a weighted average of the relative current ingestion rates which result from feeding in each mode. Within each feeding mode, WET allows two ways of determining the diet composition: indiscriminate or optimal. For indiscriminate feeding, all potential prey items are included in the diet in proportion to their availability, regardless of any differences in food quality (*f*_gut_, see previous section). For optimal feeding, the diet composition varies in time, and is calculated in such a way as to maximize the assimilated energy in each time step; this may entail excluding abundant food types of low quality (high *f*_gut_), when other, more profitable food types are available. Diet composition was set to optimal for all model runs in this manuscript.

### 2.3 The Water Ecosystems Tool (WET) model setup and nutrient enrichment scenarios

To investigate how dynamic descriptions of animal interactions can improve complex ecosystem models, the optimal behavior model formulated in the previous sections was implemented in the fish module in the process‐based, end‐to‐end aquatic ecosystem model Water Ecosystems Tool (WET) version 2.0 (DOI: 10.5281/zenodo.8369208). The WET model accounts for mass balances of nitrogen, phosphorous and silicon in a lake ecosystem with organic (dissolved and particulate organic matter) and inorganic nutrients. The model is fully modularized, featuring modules describing phytoplankton, submerged rooted macrophytes, zooplankton, detritivorous macrozoobenthos, and fish that can be individually turned on or off, duplicated and configured at runtime (Schnedler‐Meyer et al., 2022). The model is one of the few widely applied, state‐of‐the‐art aquatic ecosystem models that includes fish communities and has been parameterized for many different lakes (e.g., Andersen et al., 2022; Kong et al., 2022; Regev et al., 2023).

We chose the eutrophic, summer‐stratified Lake Bryrup, Denmark, as a testcase for the model. The lake has a mean depth of 4.6 m and a maximum depth of 9 m with summer mean total nitrogen and phosphorus concentrations ranging between 1.5–4.0 and 0.03–0.14 mg L^−1^, respectively, and chlorophyll *a* concentrations between 39 and 83 μg/L in the period 1990 to 2010. A model setup by Chen et al. (2020), which used a previous version of WET (named FABM‐PCLake) coupled to the one‐dimensional water column model General Ocean Turbulence Model lake branch (https://gitlab.com/WET/gotm/‐/tree/au) through the Framework for Aquatic Biogeochemical Models (Bruggeman & Bolding, 2014), excellently reproduced seasonal variations and magnitudes in water temperature, dissolved oxygen, N‐ and P‐dynamics as well as phytoplankton community shifts across a 10 year period. However, their model study simulated very low to extinct fish biomasses. Therefore, we modified their model study to account for a more representative fish community and biomasses by modeling two fish groups: Omnivorous fish (feeding on zooplankton, benthos, and organic material in sediment) and predator fish (feeding on the omnivorous fish), and reparametrized the two fish groups, see Appendix S2 for parameter values for the two fish groups.

To exclude transient behaviors and interannual environmental variability from the model results, we looped 1 year's forcing (1990) of weather and nutrient input for a 100‐year period. This setup was then used to test different model parameterizations, and the output from the last 50 years of model simulations was extracted from each model run and averaged over the water column using means weighted by model layer volume or bottom area, as relevant.

We also tested the response of the control model, as well as of the model with the OBM, along a trophic gradient. Five nutrient loading scenarios were created by varying the nitrogen and phosphorus load with 25%, 50%, 100%, 200%, or 400% of the baseline scenario. The nutrient load response was then compared to the trophic structure in 71 Danish lakes along a phosphorus gradient (Jeppesen et al., 2000, Figure 3).

## 3 RESULTS

### 3.1 Emergent functional response

**FIGURE 2:**
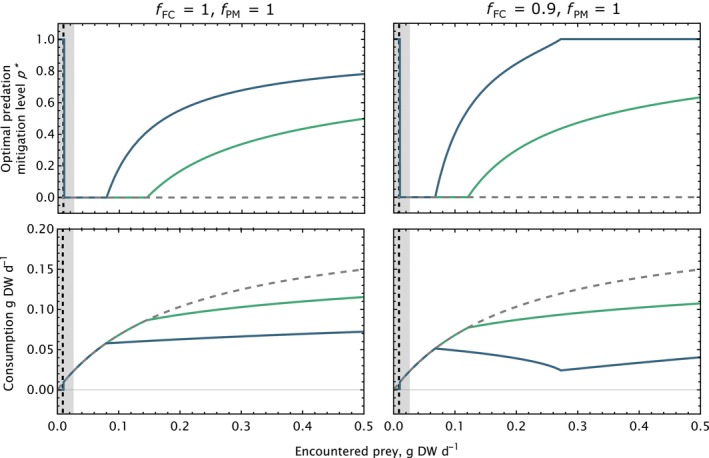
Optimal predation mitigation level and feeding as a function of scaled food availability (encountered prey over maximum consumption), for high (blue lines) and low (green lines) predation (*v*_Pred.max_ = 0.001 and 0.01 d^−1^, respectively). Gray dashed line represents constant maximized feeding (Holling type II). Top row: Optimal level of predation mitigation p* as a function of food availability (encountered food scaled to max consumption). Bottom row: Emergent functional response for scaled consumption (actual consumption over maximum possibly consumption). Shaded area: area where growth rates are negative. Dashed vertical line: Resource threshold below which feeding metabolisms exceed energy gain from feeding. Left column: *f*_FC_ and *f*_PM_ = 1. Right column: *f*_FC_ = 0.9 and *f*_PM_ = 1.

Figure 2 shows how the optimal position along the behavioral gradient, as characterized by the value of p*, changes with increasing food availability, for two different values of ambient predation risk (green and blue lines), and for both maximal and submaximal values of predation mitigation feeding cost (left and right, respectively). Note that when predation mitigation is fully effective (*f*_PM_ = 1), as in this figure, the inverse of this curve shows the vulnerability of the fish to predation. Generally, as food availability increases, the ever‐diminishing returns of increased feeding reaches a point where it becomes more advantageous for the fish to employ cost‐minimizing behaviors (i.e., increase *p*). At this point, the consumption is reduced relative to that predicted by the standard type II functional response (dashed gray curves; Figure 2, bottom row). When ambient predation risk is increased, this point is reached at a lower food availability, meaning that increased feeding is traded off against the increased mortality, and thus, the functional response is lowered. When food is scarce (below the vertical dashed line), the fish stops feeding altogether to preserve energy. In the case where feeding is impossible for maximized predation mitigation (i.e., *f*_FC_ = 1), the net result of optimizing *p* is the emergence of a functional response that shares features with the classic Holling type III functional response (Figure 2, left). Note, however, that this emergent functional response is dynamic, and changes shape depending on ambient predation risk. A more complex pattern is observed when *f*_FC_ takes values below 1 (Figure 2, right column). In these cases, increasing *p* comes at a lower cost, and fish therefore start employing risk mitigation behaviors at a lower food availability. Furthermore, when predation is high, predation mortality decreases faster than consumption with increased predation mitigation, and p* therefore rapidly rises with increased food availability until p* reaches a value of one. This has the consequence that the functional response is actually predicted to decrease for intermediate levels of food availability (Abrams, 1989). Once the optimal level of predation mitigation reaches maximum, consumption increases again, albeit from a much‐reduced level, compared to the low predation case (Figure 2, lower right panel).

### 3.2 General model behavior and stabilization of dynamics

**FIGURE 3:**
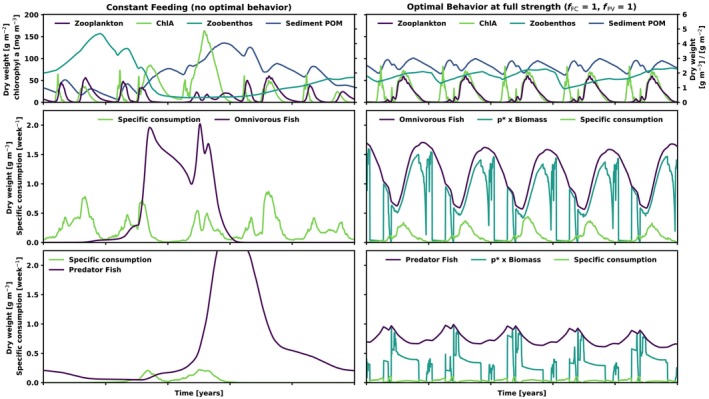
Simulated dynamics of the modeled lake food web, for a simulation with looped seasonal forcing, with (left) and without (right) the optimal behavior model (OBM). All values are water column averages, weighted by layer volume or bottom area, as appropriate. Top row: Concentrations of sediment particulate organic matter (POM) and chlorophyll *a* (ChlA; both left *y*‐axis), as well as biomass concentrations of zoobenthos and zooplankton (right *y*‐axis). Middle row: Biomass concentration and specific total consumption of omnivorous fish. In the right panel, the part of the biomass proportionate to p* is also shown (i.e., the part of the biomass that is unavailable for predation when $f_{\mathrm{PM}}$ = 1). Bottom row: Same as above, but for predator fish. With constant feeding, predator fish biomass reaches a peak of 3.16 g m^−3^.

Without the optimal behavior model (OBM), the model exhibits multiyear cyclic behavior with a 5‐year frequency and high amplitudes, even in the absence of between‐year variability in the forcing (Figure 3, left). This was a result of manually calibrating the original model calibration by Chen et al. (2020) highlighting the challenge of calibrating the default WET model to accurately capture nutrient and phytoplankton dynamics, while also simulating realistic fish biomass concentrations. The pattern is driven by a mismatch between fast growth rates reached by both fish when prey is abundant (note how specific consumption is generally high for omnivorous fish), and the comparatively slow decline in predator abundance when omnivorous fish biomass is low. The resulting cycles in fish biomass cascade down the food web, inducing a (less extreme) 5‐year cycle in the lower trophic levels. In contrast, when the OBM is switched on (trade‐off at maximum intensity, i.e., *f*_PM_ and *f*_FC_ = 1), the amplitude of the multiyear cycle is reduced dramatically, at all trophic levels (Figure 3, right).

Generally, both omnivorous and predator feeding effort is highest during late winter/early spring, when food resources are low for omnivorous fish, which are therefore forced to reduce predation mitigation in order to ward off starvation (see Figure 2). This is reflected in the low consumption of omnivorous fish in this period (Figure 3, center right). For predator fish, the low predation mitigation of omnivorous fish during this time of year results in their consumption being concentrated in this period. In contrast, the summer and fall period is characterized by high omnivorous consumption, and high levels of predation mitigation, resulting in low consumption and feeding effort of predator fish. Although omnivorous fish biomass is high and increasing during this time, prey availability is low, as indicated by the area between the biomass and p* times biomass curves in Figure 3, center right.

The consumption by omnivorous fish is thus more seasonally distributed and overall lower than in the model without OBM, in accordance with the observations regarding the emergent functional response (Figure 2). The high level of predation mitigation during times of high food availability slows down the resulting increase in biomass, while also limiting the trophic transfer of high summer production through to the predator fish. For the predator fish, the ability to reduce metabolism during period of low prey availability (as indicated by high predator fish p* values during summer) dampens their decline in biomass during starvation. These mechanisms contribute to the stabilizing effect of the OBM, which compensates for slow biomass responses with fast adjustments in p*.

### 3.3 Effect of trade‐off strength on model stability and trophic structure

**FIGURE 4:**
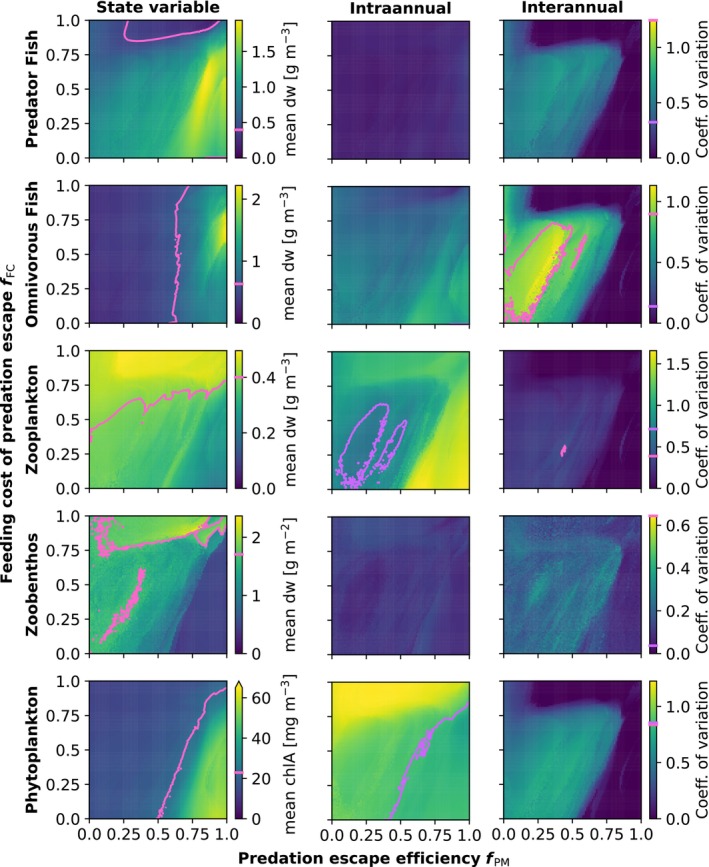
Stable dynamics of key food web elements, for different parametrizations of the omnivorous fish maximum effectiveness of predation mitigation $f_{\mathrm{PM}}$ and feeding cost of predation mitigation $f_{\mathrm{FC}}$. Left column: Mean biomass (chlorophyll *a* for phytoplankton) as averaged over the last 50 years of a 100‐year simulation with looped forcing. Middle column: Within‐year coefficient of variation, calculated from the days of the average yearly cycle. Right column: Between‐year coefficient of variation, calculated from the annual means. Note different color bar scaling between food web elements. For comparison, isolines representing the control model solution without optimal behavior are drawn in pink and purple, and their values indicated on the color bars.

Figure 4 shows the mean values, as well as the between‐year and within‐year coefficients of variation, for water column averages of selected food web elements as a function of omnivorous fish maximum effectiveness (*f*_PM_) and feeding cost of predation mitigation (*f*_FC_). The values were obtained from the last 50 years of a 100‐year simulation, forced with a constant yearly cycle of weather and nutrient input. As predator fish are top predators in this model, their optimal value of p* depend only on energetic considerations which are not influenced by trade‐off strength, and so for predator fish, the trade‐off is reduced to a question of optimizing net energy gain. We did therefore not vary *f*_PM_ and *f*_FC_ for the piscivore throughout our simulations, and all treatment of these two parameters in the rest of this section will therefore apply to omnivorous fish *f*_PM_ and *f*_FC_.

The biomasses predicted by the different parameterizations of the OBM fall within realistic ranges, when compared to data from temperate lakes (Jeppesen et al., 2000) and vary around the values predicted by the control model (Figure 4). Within this variation, however, varying the strength of the feeding trade‐off through manipulation of the cost and effectiveness of predation mitigation has a profound impact on model trophic structure. Overall, the food web structures predicted exhibit trophic cascade‐type patterns characterized by alternating high and low biomasses down through the trophic levels. However, depending on the trade‐off strength, these food web structures vary along a gradient of trophic cascade intensity and direction. A large region of relatively unstable solutions characterized by low‐intermediate biomasses across the food web and dominated by primary consumers and predator fish exists for low‐to‐intermediate cost and effectiveness of predation mitigation. In the region where predation mitigation is expensive, the food web is generally dominated by the primary consumers. Here, the total fish biomass is low, and shifts from being dominated by the top predator at low predation mitigation effectiveness, to gradually becoming more dominated by omnivorous fish as the effectiveness of predation mitigation (*f*_PM_) increases. In the region where predation mitigation is highly effective, the solution is generally dominated by fish and phytoplankton, except for a transitional area at high predation mitigation cost. Here, the simulated fish biomass is dominated by two trends; a humped relationship between trade‐off strength and total fish biomass which peaks at intermediate values of especially predation mitigation cost, and a gradual shift from top predator dominance to omnivorous fish dominance as predation mitigation effectiveness increases.

To analyze model stability, we separated the contributions of between‐year and within‐year variation by calculating the coefficient of variation for both the yearly means and an average year of selected food web elements (Figure 4, intra‐ and interannual variation columns). In general, under a stable seasonal forcing regime, relative large seasonal cycles are expected for especially the lower trophic levels in contrast with large‐amplitude multiyear cycles. For predator fish and zoobenthos, within‐year coefficients of variation are similar to the control model, and are generally less influenced by OBM trade‐off strength compared to between‐year variability, whereas within‐year variation was higher than the control model for large regions of trade‐off parameters space for phytoplankton, zooplankton, and omnivorous fish. However, these increases in within‐year variability reflects a much more realistic seasonal cycle in the case of the plankton, while this increase for omnivorous fish should be seen in the context of the fish biomass dynamics of the control solution, which is characterized by several years where the biomass is continuously very low, thus reducing average within‐year variation.

In contrast, the stabilizing effect of the OBM on between‐year variation is shown to be a robust feature of the OBM, lowering between‐year variation for most parameter combinations, and almost eliminating them in a large region where predation mitigation is costly and/or efficient. Outside of this region, the solution is characterized by multiyear cyclic behaviors that are generally less drastic compared to the control model, except for omnivorous fish biomass when predation mitigation is of intermediate cost and low efficiency.

To test for the importance of the optimal diet breadth for the results presented here, we reran the model simulations presented in Figure 4 with fixed diets (not shown). Generally, diet optimization contributes only slightly to the stabilization of the model and only has minor displacing effects of the biomass patterns of Figure 4. Exception where omnivorous fish, which had somewhat lower biomass throughout, and zoobenthos, the biomass of which now followed the chlorophyll *a* concentration, in contrast with the pattern of the simulations with optimal diet breadth (Figure 4). These changes are unsurprising, as with a fixed diet the bentivorous feeding tend to be dominated by POM consumption, which has a high *f*_*gut*_ value (see Appendix S2: Fish parameter values).

### 3.4 Nutrient loading scenarios and intraspecific interference

**FIGURE 5:**
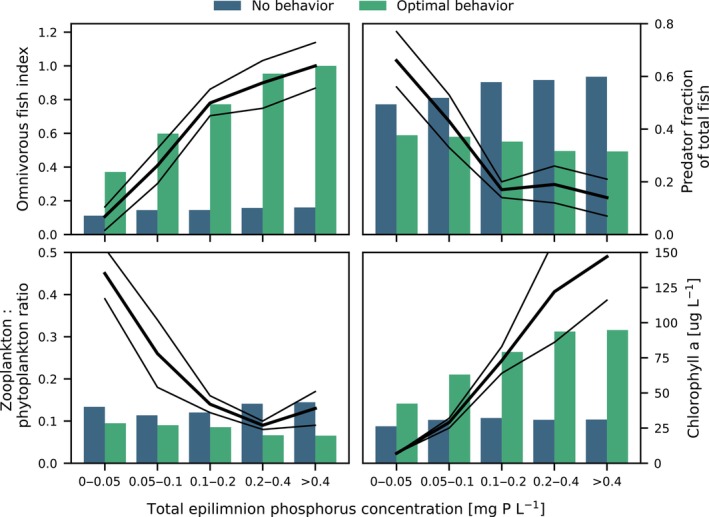
Model response to nutrient enrichment scenarios compared to data (black lines, mean ± SD), with and without the optimal behavior model (OBM); No behavior: OBM turned off. Optimal behavior: Optimal behavior turned on for both omnivorous ($f_{\mathrm{PM}}$ = 0.9, $f_{\mathrm{FC}}$ = 0.675) and predator fish ($f_{\mathrm{PM}}$, $f_{\mathrm{FC}}$ = 1). Top left: Index of Mean August omnivorous fish biomass, normalized to the maximum value across the two configurations. Top right: Mean August predator fraction of total fish biomass. Bottom left: Mean summer (1 May–1 October) zooplankton to phytoplankton biomass ratio. Bottom right: Mean summer epilimnion chlorophyll *a* concentration. Metrics were chosen to match the data from Jeppesen et al. (2000, black lines).

We compare the ability of the model with and without the OBM to reproduce general patterns in lake ecosystem response to nutrient loading. Figure 5 displays time‐ and space‐averaged selected food web metrics of the model along a nutrient loading gradient, compared with empirical patterns in the same metrics from 71 Danish lakes along a eutrophication gradient (Jeppesen et al., 2000; Figure 5, black lines). For the model configuration with optimal behavior, we chose a set of OBM parameters for which the resulting food web structure (see Figure 4) corresponded relatively well with empirical observations from temperate lakes of the same baseline nutrient loading (100% scenario, represented by the 0.1–0.2 mg P L^−1^ tick on the *x*‐axis of Figure 5).

The control model had a muted response to nutrient enhancement, all food web metrics varying relatively little with nutrient level, as compared to the empirical pattern. This model configuration was dominated by a strong top‐down effect of the piscivore, resulting in underestimation and overestimation, respectively, of omnivorous and predator fish biomasses for all but the lowest nutrient loadings. Similarly, summer mean chlorophyll *a* concentration did not increase much with nutrient availability.

Overall, the data were better represented by the model configuration that included optimal behavior. The chosen trade‐off configuration (*f*_PM_ = 0.9 and *f*_FC_ = 0.675 for omnivorous fish) of the model with OBM represented a region in Figure 4 where omnivorous fish risk mitigation is efficient and of intermediate cost, and omnivorous fish were therefore partly released from trophic control. Consequently, the model is much more responsive to nutrient loading overall, with an overall better correspondence with the empirical pattern. Across nutrient levels, the responses of all food web responses matched the signs of the corresponding empirical trends, while both the omnivorous fish indicator as well as chlorophyll *a* also matched the magnitudes quite well. The loosened trophic control on omnivorous fish cascades through the zooplankton and allows chlorophyll *a* to generally increase with nutrient forcing.

## 4 DISCUSSION

We implemented an Optimal Behavior Model (OBM) in a complex ecosystem model, using a generalized and intentionally simplified approach, and showed how consideration of the trade‐off between feeding, energy expenditure, and predation leads to (1) stabilization of the food web dynamics, most notably through the dampening of unrealistic long‐period oscillations in fish biomass, (2) a variety of food web structures that emerges based on the trade‐off strength, and (3) food web responses to nutrient forcing that are more aligned with observations.

Historically, it has been difficult to calibrate WET and its predecessor to exhibit realistic fish biomasses, yielding unrealistic responses of lower food web elements to environmental forcing (T.K. Andersen, Dennis Trolle, pers. comm.). The particular calibration used as a starting point for this study predicted realistic chlorophyll *a* responses to nutrient enhancement (Chen et al., 2020), but was not able to sustain fish populations in its original form. In the control version of the model from this study, fish were manually calibrated to be present, even in the absence of the OBM, but this induced strong top‐down regulation in the model, generating the large‐amplitude, low‐frequency cycling seen in Figure 3 (left panels) and preventing the model from responding realistically to the nutrient enrichment scenarios (Figure 5). With optimal behavior, the model was able to simulate both realistic levels of fish biomass, while still reproducing observed seasonality and magnitude of phytoplankton as in Chen et al. (2020). Here, we have demonstrated a relatively simple, yet flexible way of improving fish descriptions to not only reflect actual behavior more accurately, but also allowing fish to reach realistic biomasses, while still allowing a realistic response to environmental forcing.

### 4.1 Stabilization of the model

Allowing growth to be traded off against mortality dynamically regulates trophic control and helps balance fast and slow ecological processes, slowing down growth under food‐replete conditions while cushioning populations from excessive predation and mitigating starvation, and we show how these effects combine to stabilize the food web dynamics. The main stabilizing effect of the OBM is to dampen the multiyear large amplitude cycles of the control model, and thus allowing for a realistic seasonal cycle of the lower trophic levels. These dynamic properties arise from the emergent functional response, which responds to changes in predation risk. The stabilizing effects of optimal behavioral responses and similar emergent functional responses on predator prey dynamics has been demonstrated before, for example, in habitat selection games (Abrams, 2007), in tri‐trophic food webs with or without intra‐guild predation (Frølich et al., 2022; Li et al., 2023; Visser et al., 2012), and for simple Lotka–Volterra systems (Křivan, 2007). Here, we extend these findings to a much more complex food web (Figures 3 and 4), in a way that is computationally efficient, and therefore relevant for complex ecological models. The OBM therefore offers a way to stabilize complex food web models rooted in mechanisms at the level of the individual, as an alternative to more traditional approaches like carrying capacities, quadratic mortality terms, and Holling type III functional responses, which all lack direct mechanistic interpretations and are consequently hard to demonstrate or quantify empirically (Geritz & Kisdi, 2012).

### 4.2 Regulation of food web structure through cascading behavioral effects

Aside from stabilizing model dynamics, our OBM implementation causes cascading effects on the food web structure that offer interesting implications for ecosystem modeling. Allowing animals to adaptively change feeding behavior in response to predation has several effects that extend to other trophic levels. First, by converting predation mortality into reduced growth, increasing the predation mitigation has the immediate effect of reduced mortality on the lower trophic levels, while also having a feedback effect on the higher trophic level through reduced predator growth. Second, when prey responds to higher predator biomass with increased predation mitigation, a density‐dependence is introduced into the predator functional response. These effects lead to apparent linkages between trophic levels that are not adjacent, which contributes in driving the variation in trophic structure simulated by the model (Figure 4).

The regulation of the trade‐off strength through the parameters *f*_PM_ and *f*_FC_, is a new development from the work of Kiørboe et al. (2017) allowing the model to generate a continuum of food web structures simply by varying the strength of the predation‐feeding trade‐off for the intermediate predator. This food web variability emerges as a result of changing trade‐off strength (*f*_FC_ and *f*_PM_) and in the absence of any changes in system forcing, productivity or intrinsic functional responses, and is similar in range to the observed variation in similar natural ecosystems (Jeppesen et al., 1997, 2000; Scheffer & Jeppesen, 2007). Here, we have assumed linear relationships between predation mitigation and its cost and effectiveness. Many other such relationships could be speculated, for example, diminishing returns or increased cost of predation mitigation with increasing levels of predation mitigation. The effects of relaxing this assumption of linearity are a subject for future studies, but we speculate that the effect of such changes would primarily be to change the relative sizes of the different biomass regions of Figure 4, and not the overall qualitative pattern. In any case, little is known about the shape of the relationship between feeding activity and predator mitigation, and so keeping it to the most basic form is arguably preferable.

The modulation of the fish group interactions and resulting food web structure introduced by the inclusion of optimized feeding are reflective of empirical observations from numerous studies on the importance of flexible trait responses like risk mitigation behavior for food web dynamics (the so‐called behavioral cascade, see Peacor et al., 2020; Preisser et al., 2005; Romare & Hansson, 2003; Schmitz et al., 2008; Werner & Peacor, 2003). Such flexible trait responses have indirect effects on trophic structure and control that are often comparable in magnitude or exceeding those of direct numerical (consumptive) responses (Schmitz, 1998; Werner & Peacor, 2003), opposite in direction to consumptive responses (Schmitz et al., 2008) or which may attenuate less through food webs (Preisser et al., 2005).

The model predicts that expensive and less‐than‐perfect predation mitigation of the intermediate predator (here omnivorous fish) should lead to a state characterized by tight trophic control, in which the top predator is able to control the prey population. This results in relatively low abundances of both top and intermediate predators and a trophic cascade of alternate high and low abundances down the food web. Alternatively, when predation mitigation is highly efficient and not too expensive, biomasses of both intermediate and top predators are high, and the resulting trophic cascade is reversed. That we were able to generate such rich dynamics through manipulation of a single trade‐off speaks to the potential benefits of considering such flexible trait responses in ecosystem model containing large animals.

The predicted food web structure resulting from efficient and cheap (expensive and inefficient) risk mitigation is similar to that typical of eutrophic (oligotrophic) temperate lake systems (Figure 4). Which real‐world environmental conditions across lake morphologies and eutrophication gradients might affect the feeding‐predation trade‐off in a particular direction is not immediately apparent, although it can be said with confidence that risk mitigation strategies available to fish do vary between lakes. Lake fish populations are known to seasonally partially migrate into streams in the winter, a behavior that is well predicted by bioenergetics considerations and individual predation risk (Brönmark et al., 2008; Skov et al., 2011), and which have been predicted to influence the dynamics of lower trophic levels (Brodersen, Ådahl, et al., 2008). Diel vertical migration in deep lakes is another example of a well‐studied risk mitigation strategy, for which the trade‐off and associated costs and benefits are well understood (Dini & Carpenter, 1992; Lambert, 1989; Mehner, 2012; Ohman, 1990; Sainmont et al., 2013). In shallow lakes, a similar diel migration might be facilitated by dense macrophyte beds (Burks et al., 2002; Romare & Hansson, 2003; Turner & Mittelbach, 1990), but the availability of macrophytes depend on lake turbidity being sufficiently low, and the associated costs and benefits are not as clear, as well as being highly species‐specific (Diehl, 1988; Persson, 1993). Meanwhile, the turbid waters of eutrophic lakes may increase the efficiency of reduced activity or schooling, or might decrease the feeding costs of such strategies, as zooplankton have less structured refuge habitat available (Diehl, 1988; Jeppesen et al., 1997). More or less universally, lake fish exhibit diel variation in activity levels (e.g., Baktoft et al., 2012; Helfman, 1981; Jacobsen et al., 2015). Such high daily fluctuations in activity level implies a relatively high cost in overall feeding but is a predation mitigation strategy that is available in all environments. The different strategies of reducing predation risk available to prey fish are therefore expected to vary in their cost and efficiency along environmental gradients like lake productivity, but whether their indirect effects line up to amplify or dampen the direct effects of enrichment might vary from case to case. This match or mismatch between direct and indirect top‐down effects offer an important dimension to studies of trophic relationships and dynamics, and might help explain the considerable variation in lake responses to the same environmental impacts (Jeppesen et al., 2000). Such a dependence of lake response on predation mitigation cost and effectiveness was also predicted by our model. To investigate the robustness of the improved model response to nutrient forcing for combinations of trade‐off parameters other than the one shown (Figure 5), we ran new parameter sweeps similar to those of Figure 4, but for the lowest and highest nutrient scenarios of Figure 5, and report on the results in Appendix S3. Generally, the response of the model to nutrient forcing varied considerably with the trade‐off parameterization. However, when we restricted the considered parameterizations to those which produce system‐appropriate metric values in the 100% scenario, we reproduced the overall patterns of Figure 5, with some variation.

Our findings therefore serve as a call for further empirical studies on how environmental factors influence and control the trade‐offs involved in trophic interactions (Abrams, 2022).

### 4.3 Generality of model approach

The model adopted here is deliberately agnostic to the specific risk‐reduction strategies employed by the prey species. Other, more specific models of similar ecosystems have focused on single strategies like refuge habitat (Genkai‐Kato, 2007), while our goal here was to retain model generality and robustness of interpretation in the face of the plethora of possible responses of lake fish to predation, many of which are poorly understood, or insufficiently quantified. A drawback of our model, however, is that this approach limits the range of indirect effects of predation in our model to the regulation of prey‐specific predator feeding efficiency and overall prey feeding efficiency. Other nonconsumptive effects are certainly operating in nature, including, but not limited to, diet shifts of the focal prey (Persson & Eklov, 1995; Schmitz et al., 2008), or responses that decreases exposure to a specific predator, while increasing exposure to others (Byrnes et al., 2006; Fiksen et al., 2005). While such effects might be important in shunting production between alternate food web pathways, and therefore, have potentially far‐reaching consequences for ecosystem function, accounting for all such potential effects requires detailed knowledge of species interactions that are often lacking. Furthermore, such an accounting would complicate already complex ecosystem models extensively, and make model configuration, parametrization, and interpretation nigh on impossible. These limitations of our model nevertheless render it most suitable for models of relatively simple food chains, and less suitable for modeling indirect effects on community structure. We argue that the present level of complexity is appropriate for models that are primarily concerned with describing ecosystem functions and properties such as nutrient cycling, trophic transfer, and water clarity, like WET. For now, and from a modeling perspective, varying the trade‐off strength can offer a useful tuning parameter for calibration of lake models, and elucidating if and how these effects vary systematically along gradients of environmental change offer an important avenue of model improvement, not only for lake models, but also indeed for ecosystem models in general.

### 4.4 Comparison with other approaches to optimal behavior modeling

Optimal (or adaptive) behavior have been implemented in models in various ways reflecting different model systems, focal processes, levels of realism and complexity (e.g., Abrams, 2007; Frølich et al., 2022; Kiørboe et al., 2017; Křivan, 2007; Li et al., 2023; Mariani et al., 2013; Pinti et al., 2021; Visser et al., 2012), and simplifying assumption are often made in order to reduce dimensionality and ease calculation of the optimal behavior (Frølich et al., 2022). A benefit of our approach is that it assumes nonlinear fitness criterions for both predator and prey, through the satiating functional responses. Many earlier or more theoretical models (Cressman et al., 2004; Křivan, 2007; Malone et al., 2020) used linear fitness proxies in the form of Holling type I functional responses, which often allows for finding analytical solutions to the populations dynamics of simple predator–prey models. However, such models fail to capture nonlinear effects stemming from satiation of the predator and prey. Until recently, most models only investigated the consequences of a single optimizing organism (predator or prey), while keeping the behavior of other species fixed (e.g., Fiksen et al., 2005; Malone et al., 2020; Mariani et al., 2013; Visser et al., 2012). This study models predator–prey coadaptation with only two organism groups for the purpose of model demonstration. As for the predator fish in our model system, the absence of a top predation reduces their foraging optimization to one that maximizes net energy gain. We could have limited our analysis to one optimizing fish group; however, we wanted to emphasize how our formulation allows many more fish groups that simultaneously adjust their behavior within the modular WET model and is computationally efficient enough to support such implementations. Simulations without predator behavior (not shown here) still result in drastically improved model behavior, while the modeled biomasses change in some regions, but not in others. We could also have chosen to model a tri‐trophic fish community, with a top predator preying on the predator fish, but feel that the present model configuration represents a good compromise of model complexity.

Here, we have chosen to model optimal behavior as a monomorphic process, meaning that the entire population behaves identically in each instant. This can lead to unrealistically high fitness, compared to the case where individuals independently optimize their fitness (Abrams et al., 1993; Frølich et al., 2022). When individuals act independently, the ensuing game leads to a Nash equilibrium where all individuals follow identical strategies, but which might be suboptimal, compared to the monomorphic case (Frølich et al., 2022). Recently, Frølich et al. (2022) successfully modeled a tri‐trophic food chain with both consumer and predator individuals independently optimizing their behavior using a numerical scheme to solve for the Nash equilibrium in each time step. It would be of interest to compare how much the predictions of the present model would change using an approach similar to theirs. While the authors claim that their numerical approach is scalable and computationally efficient, we wonder whether such an approach is really feasible in a spatially resolved model like WET that is often run hundreds of thousands of times during model calibration.

### 4.5 Robustness of the chosen fitness criterion

A fundamental assumption in our model is that the measure of food intake over mortality as dictated by current conditions is a good proxy for the long‐term fitness of organisms (i.e., Gilliam's rule, Fraser & Gilliam, 1987). While Gilliam's rule normally assumes a stable environment, Sainmont et al. (2015) showed that this so‐called myopic (or short‐sighted) fitness measure can be a good approximation for lifetime fitness, even in strongly seasonal environments. Nevertheless, the possibility of storing energy reserves for lean times might change the predicted optimal behavior somewhat. In seasonal environments, many fish buildup reserves during summer, reserves that are partly utilized for reproduction in spring, but are used during times of low food availability in the winter season. As can be seen in Figure 3, in spite of higher consumption during the summer, our model predicts that foraging effort is highest during the meager winter months. However, if energy storage is taken into account, it might be optimal to concentrate feeding in the summer, while utilizing stored resources to reduce predation risk during winter. Indeed, the high occurrence of partial migration of lake fish during winter is an example of this (Brönmark et al., 2008; Skov et al., 2011). Our results fits well with observations of the winter behavior of fish in low condition, which are less likely to engage in winter partial migrations (Brodersen, Nilsson, et al., 2008). The inclusion of energy storage would likely result in more seasonal synchronization of fish consumption, which would function to reduce seasonal amplitudes of fish biomass even more.

### 4.6 Future perspectives

The chosen model setup represents a simplified representation of a temperate lake ecosystem and its fish community. In order to investigate the impact of added ecological realism, several avenues of increased complexity are available for future investigation. Adding more life stages to the modeled fish populations in order to investigate effects such as cannibalism on early life stages and competition between prey fish and early life stages of predators would be a natural step to investigate the robustness of the present findings (Arranz et al., 2016; Claessen et al., 2000; Rudolf, 2007, 2008). Another interesting line of investigation would be how the OBM might contribute to reproducing fish community succession patterns along gradients in environmental forcing (Arranz et al., 2016; González‐Bergonzoni et al., 2012; Jeppesen et al., 2000, 2010; Persson & Hansson, 1999).

Numerous studies have demonstrated that zooplankton employ complex predator avoidance behaviors in both marine and lake environments, including diel vertical migrations to depth, as well as horizontal migration to refuge habitat such as macrophyte beds (Burks et al., 2002; Dini & Carpenter, 1992; Hays, 2002; Lambert, 1989). So far, we have not tested the inclusion of behavior to the zooplankton module of WET. In our simulations, zooplankton to phytoplankton biomass ratio was one of the least well‐represented ecosystem metrics (see Figure 5). It appears likely that allowing zooplankton to adapt their feeding effort to changing predator and food landscapes would improve their response to system productivity, similarly to how it was improved for omnivorous fish.

To the best of our knowledge, this study represents the first time that the effect of optimal behavior of large animals within a complex end‐to‐end ecosystem model has been investigated. Here, we demonstrate that such an approach is indeed feasible and offers tangible benefits for model performance. Additionally, we have outlined a few lines of investigation that are promising for future studies. We conclude that a simplified approach like the one employed here offers promising possibilities for improving ecosystem modeling in general, especially when it comes to the (often lacking) representation of large animals in biogeochemical and ecological models.

## AUTHOR CONTRIBUTIONS

**Nicolas A. Schnedler‐Meyer:** Conceptualization (equal); formal analysis (lead); investigation (lead); methodology (lead); software (lead); validation (equal); visualization (lead); writing – original draft (lead); writing – review and editing (equal). **Tobias K. Andersen:** Conceptualization (equal); formal analysis (supporting); investigation (supporting); methodology (supporting); software (supporting); validation (equal); writing – original draft (supporting); writing – review and editing (equal).

## Supporting information


Appendices S1–S3.

## Data Availability

The latest stable WET version used throughout this work has been released through zenodo: https://zenodo.org/records/8369208. The WET source code as well as instructions on how to compile with gotm‐lake can be obtained through the WET website at https://projects.au.dk/wet.
