# Supplementary material for: Dining in danger: Resolving adaptive fish behavior increases realism of modeled ecosystem dynamics
Source: Ecol Evol. 2024 Aug 7;14(8):e70020. doi: 10.1002/ece3.70020 (PMC11303985; doi:10.1002/ece3.70020)
Supplement: Supplementary file 1 — Appendices S1–S3. [file ECE3-14-e70020-s001.docx]

**Appendix S1:**

**Detailed description of fish feeding in the Water Ecosystems Tool (WET)**

*Feeding rate and Optimal Diet Breadth (ODB)*

The WET fish module has access to three basic feeding modes (*j = 1, 2, 3*): Benthivory, planktivory and piscivory. Each feeding mode include a number of prey classes, *i,* each with gut occupation factor, $f_{gut.ij}$ (higher for prey of low digestibility or energy density), representing energy density and digestive ease of prey *i* of feeding mode *j* (see Visser and Fiksen, 2013).

WET contains two methods of determining diet composition based on available prey: indiscriminate (or opportunistic) feeding, and Optimal Diet Breadth (ODB). In the first case, prey is simply ingested in the proportions encountered, in line with previous versions of the model, and there is no active selection between different prey. For both indiscriminate and optimal feeding, the feeding (or satiation) level $F_{j}$ resulting from exclusive feeding in mode *j* is calculated, based on the prey concentrations or densities $R_{i}$ (*i* = 1, 2, …, *n*) in that mode (Sainmont et al., 2013):

$$F_{j}= \frac{f_{Veg.j} \sum_{i,j} \left( c_{i,j}f_{gut.i,j}R_{i,j} \right)}{I_{max}(T)+ f_{veg.j}\sum_{i} \left( c_{i,j}f_{gut.i,j}R_{i,j} \right)}$$

(A1)

where $c_{i,j}$ is the maximum clearance rate on prey *i* in feeding mode *j*, $I_{max}(T)$ is (temperature-dependent) maximum (gut-limited) consumption, and $f_{veg.j}$ is the (optional) impact of vegetation on catchability. Note that maximum consumption is equal for all feeding modes.

Potential consumption of prey *i* in feeding mode $j$ is then calculated as total encountered and caught prey, times the ‘hunger level’ (one minus the feeding level).

$$I_{pot.i,j}=f_{Veg.j}c_{i,j}R_{i,j}\left( 1-F_{j} \right)$$

(A2)

and total potential consumption in feeding mode $j$ is then

$$I_{pot.j}=\sum_{i=1}^{n} I_{pot.i,j}$$

(A3)

The optimal diet breadth for a given prey field is the set of prey classes that maximize energy intake at any given moment. Because prey have varying gut occupation factors, including prey with low energy density might not be optimal, when gut space or search time can instead be spent on prey that is more profitable. Thus, the decision to include a prey type at any given time depends in part on its profitability, and in part on the availability of other prey (Visser and Fiksen, 2013). Prey profitability is calculated as

$$p_{i,j}=\frac{1}{f_{gut.i,j}}$$

(A4)

To calculate ODB, prey must first be ranked according to their profitability, and consumption then calculated in an iterative manner, sequentially including additional prey types in order of descending profitability, until the new consumption rate resulting from adding the next prey is lower than the one resulting from its exclusion. Thus, in each feeding mode, prey types are ranked such that

$$p_{i,j}>p_{i+1,j}$$

(A5)

For a set of available prey of type *i* = 1, …, *k*, …, *n*, the optimal set of prey types in the diet is then the subset of *k* prey types for which

$$I_{pot.j}=\sum_{i=1}^{k} I_{pot.i,j}, where \sum_{i=1}^{k} I_{pot.i,j}>\sum_{i=1}^{k+1} I_{pot.i,j}$$

(A6)

Apart from variability in the included prey, feeding level and consumption is calculated in the same way as in the indiscriminate case.

*Impact of vegetation on catchability*

If relevant to the modelled case, impact of vegetation on catchability $f_{veg.j}$, of prey in each specific feeding mode $j$ is calculated as:

$$f_{veg.j}=1-q_{j}Q$$

(A7)

where $Q$ is submerged vegetation coverage in percent and $q_{j}$ is the relative impact of submerged vegetation coverage on catchability of prey for feeding mode $j$. For an example of vegetation impacting catchability of prey, see Diehl (1988).

*Feeding mode selection*

In order to calculate the contributions of each feeding mode to the total fish diet, we assume that feeding modes are separated and non-overlapping in time, and that there is no penalty in switching feeding modes. Optimally, fish should devote all their feeding effort to the feeding mode that yields the highest consumption. Here we will assume that fish have imperfect information and continuously sample the feeding modes that are available to them, but utilize them in proportion with their relative fitness contributions. Inspired by Visser and Fiksen (2013), the fraction of feeding effort fish spend in each feeding mode is equal to the consumption rate resulting from that mode as a fraction of the sum of consumption rates from all feeding modes, i.e. a weighted average:

$$E_{j}=\frac{I_{pot.j}}{\sum_{j=1}^{3} I_{pot.j}}$$

(A8)

where $E_{j}$ is the fraction of foraging effort spent on feeding mode $j$. Maximum potential assimilation in feeding mode $j$ is then:

$$g_{pot.j}=aE_{j}I_{pot,j}$$

(A9)

where $a$ is the assimilation efficiency (dimensionless). Total potential assimilation (i.e. assuming constant feeding at *p* equal to one) is then:

$$g_{pot}=\sum_{j=1}^{3} g_{pot.j}$$

(A10)

This value is then used in the calculation of *p**.

*Calculating p* in the case of multiple feeding modes and prey types*

In cases, where net potential growth is negative (i.e. $g_{pot}-u_{pot} <0$), *p** is found in the same ways as for the single prey case described in the methods section. If net growth is positive, we first calculate diet composition, feeding mode allocation and consumption assuming maximized feeding. We then calculate overall food availability, based on the included prey types and allocation of feeding effort to each feeding mode:

$$R_{diet}= \sum_{j=1}^{3} \left( E_{j}f_{Veg.j}\sum_{i=1}^{k} \left( c_{i,j}f_{gut.i,j}R_{i,j} \right) \right)$$

(A11)

We then calculate a combined overall diet $f_{gut}$ value, based on the relative contributions to the overall diet of all prey:

$$f_{gut.all}= \sum_{j=1}^{3} \left( E_{j}\sum_{i=1}^{k} \left( \frac{{f_{gut.i,j}I}_{pot.i,j}}{I_{pot.j}} \right) \right)$$

(A12)

We can then calculate *p** as in eq. 10, by substituting $R_{diet}$ and $f_{gut.all}$ in place of $R_{e}$ and $f_{gut}$, respectively.

This estimated optimal predation mitigation value is then used to calculate the final realized consumption in each feeding mode, using equations 1 and A1-3:

$$F_{j}= \frac{f_{Feed}f_{Veg.j} \sum_{i,j} \left( c_{i,j}f_{gut.i,j}R_{i,j} \right)}{I_{max}(T)+ f_{Feed}f_{veg.j}\sum_{i} \left( c_{i,j}f_{gut.i,j}R_{i,j} \right)}$$

(A13)

$$I_{j}=\sum_{i=1}^{n} {f_{Feed}f}_{Veg.j}c_{i,j}R_{i,j}\left( 1-F_{j} \right)$$

(A14)

Including $f_{Feed}$ in the determination of the optimal diet breadth could in some cases change the resulting diet composition somewhat, because values of $f_{Feed}$ below one lowers both the encounter rates with prey, as well as fish overall satiation. Both optimizations should therefore be seen as estimates of the true optimal behavior, reflecting the separation of the feeding behavior into two discrete decisions.

**Appendix S2:**

**Fish parameter values**

| **fish type** | **parameter** | **model code name** | **value range** | **unit** | **explanation** |
| --- | --- | --- | --- | --- | --- |
| **Omnivorous fish** | *p* | - | 0-1 | - | level of predation mitigation |
|  | *p** | Pstar | 0-1 | - | optimal level of predation mitigation |
|  | *f_FC_* | fFC | 0-1 | - | feeding cost of predation mitigation |
|  | *f_PM_* | fPV | 0-1 | - | predation mitigation efficiency |
|  | *f_Veg.Bent_* | aFunVegBent | 0 | - | effect of vegetation on benthivory |
|  | *f_Veg.Plan_* | aFunVegPlan | 0 | - | effect of vegetation on planktivory |
|  | *c_zoop_* | cClearPlan1 | 0.276 | m3 gDW-1 d-1 | specific clearance rate on zooplankton |
|  | *c_zoob_* | cClearBent1 | 0.047 | m2 gDW-1 d-1 | specific clearance rate on zoobenthos |
|  | *c_POM_* | cClearBent2 | 0.403 | m2 gDW-1 d-1 | specific clearance rate on sediment POM |
|  | *f_gut.zoop_* | fGutOccPlan1 | 1 | - | gut occupation factor for zooplankton |
|  | *f_gut.zoob_* | fGutOccBent1 | 1 | - | gut occupation factor for zoobenthos |
|  | *f_gut.POM_* | fGutOccBent2 | 7.2 | - | gut occupation factor for POM |
|  | *I_max_* | kDConsMax | 0.214 | d-1 | specific maximum consumption |
|  | *a* | fDAssFi | 0.8 | - | assimilation efficiency |
|  | *m_nat_* | kMortFi | 5E-04 | d-1 | specific natural mortality |
|  | *u* | kDRespFi | 1.39E-02 | d-1 | specific basic metabolism |
|  | *f_RF_* | fDRespActFi | 0.682 | - | extra metabolic feeding factor |
| **Predator fish** | *p* | - | 0-1 | - | level of predation mitigation |
|  | *p** | Pstar | 0-1 | - | optimal level of predation mitigation |
|  | *f_FC_* | fFC | 0-1 | - | feeding cost of predation mitigation |
|  | *f_PM_* | fPV | 0-1 | - | predation mitigation efficiency |
|  | *f_Veg.Pisc_* | aFunVegPisc | - | - | effect of vegetation on piscivory |
|  | *c_pisc_* | cClearPisc1 | 0.031 | m3 gDW-1 d-1 | specific clearance rate on omnivorous fish |
|  | *f_gut_* | fGutOccPisc1 | 1 | - | gut occupation factor for omnivorous fish |
|  | *I_max_* | kDConsMax | 0.0753 | d-1 | specific maximum consumption |
|  | *a* | fDAssFi | 0.9 | - | assimilation efficiency |
|  | *m_nat_* | kMortFi | 5E-04 | d-1 | specific natural mortality |
|  | *u* | kDRespFi | 5.60E-03 | d-1 | specific basic metabolism |
|  | *f_RF_* | fDRespActFi | 0.767 | - | extra metabolic feeding factor |

**Appendix S3:**

**Nutrient enrichment and trade-off strength**

Fig. A1 (left) compares food web metrics similar to those of Figure 5 across OBM trade-off parameters *f_FC_* and *f_PM_,* between the 100% nutrient scenario (Fig. 4, 5), and the minimum (20 %) and maximum (400 %) nutrient scenarios. In the left panel of Fig. A1, the change in percent of the average food web metrics is displayed for all combinations of *f_FC_* and *f_PM_.* As can be seen, the diversity of food web structures predicted by the model (Fig. 4) is reflected by a corresponding diversity in the predicted responses of the food web. As such, there is no universal response of the model to nutrient forcing, which reflects the different degrees of trophic control that results from different trade-off parameterizations. However, only a subset of all the possible parameterizations generates food web structures that are broadly in line with empirical observations from systems with nutrient regimes similar to the 100 % nutrient scenarios. When we restrict the pool of parameterizations to those that broadly conform to the expected food web structure (black lines in Fig. A1, left), we get a more constrained set of nutrient scenario responses (Fig. A1, right), which, while still containing a considerable amount of variation, broadly follows the pattern of Fig. 5.


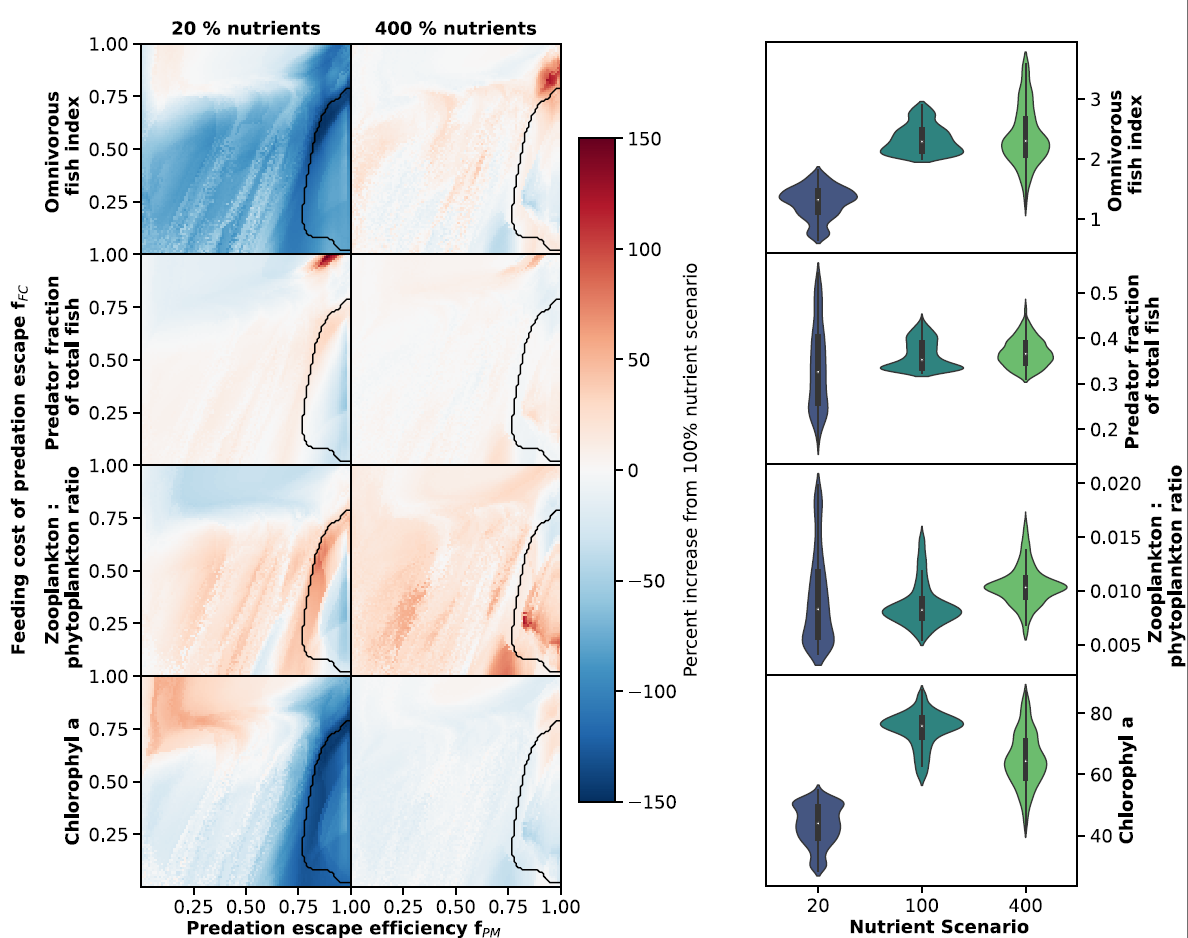


***Fig. S1. Model response to the minimum and maximum nutrient enrichment scenarios across trade-off parameterizations (effectiveness of predation mitigation*** $\boldsymbol{f}_{\boldsymbol{PM}}$ ***and feeding cost of predation mitigation*** $\boldsymbol{f}_{\boldsymbol{FC}}$***). Left: Change in food-web indicator metrics (see Fig. 5), as a percentage of the metric value at the 100% nutrient forcing scenario. Black line delineates the area where the 100% nutrient scenario predicts average summer chlorophyl* a *concentrations above 30 µg L^-1^, average omnivorous fish August biomass above 2 gDW m^-3^, and average predator fish August biomass above 0.25 gDW m^-3^. Right: Violin plots showing the distribution of predicted metrics within the bounded region of the left side panels, for the three nutrient scenarios.***
